# Supplementary material for: MetaRibo-Seq measures translation in microbiomes
Source: Nat Commun. 2020 Jun 29;11:3268. doi: 10.1038/s41467-020-17081-z (PMC7324362; doi:10.1038/s41467-020-17081-z)
Supplement: Supplementary file 10 — Supplementary Data 7 [file 41467_2020_17081_MOESM10_ESM.zip › File2/Confidence_High_Taxonomy/367928_out.krona.html]

Javascript must be enabled to view this page.

members
magnitude
magnitudeUnassigned
count
unassigned
taxon
rank

367928\_out

8

8
superkingdom
2

phylum
8
1224

class
8
1236

order
8
91347

543
8
family

8

SRS140492\_contig\_number\_30689
1
genus
561

7
species

SRS012273\_contig\_number\_8752SRS017191\_contig\_number\_19044SRS017521\_contig\_number\_31602SRS049402\_contig\_number\_16612SRS144362\_contig\_number\_42404SRS146888\_contig\_number\_11686SRS147022\_contig\_number\_14663
562
